# Tuning Physical Crosslinks in Hybrid Hydrogels for Network Structure Analysis and Mechanical Reinforcement

**DOI:** 10.3390/polym11020352

**Published:** 2019-02-18

**Authors:** Xue Lv, Chuang Liu, Zhubao Shao, Shulin Sun

**Affiliations:** Polymeric and Soft Materials Laboratory School of Chemical Engineering, Changchun University of Technology, Changchun 130012, China; pies112@126.com (X.L.); ccutliuchuang@163.com (C.L.)

**Keywords:** hybrid particles, hydrogels, mechanical properties, internal structure

## Abstract

Hydrogels with high mechanical strength are needed for a variety of industrial applications. Here, a series of hydrogels was prepared by introducing hybrid particles as hydrophobic association points to toughen the hydrogels. These toughened hydrogels were able to transfer an external mechanical force via the reorganization of the crosslinking networks. They exhibited an extraordinary mechanical performance, which was the result of the coordination between hydrophobic segments and hybrid particles. Herein, the connection between the dissipated energy of the inner distribution structure (on a small scale) and the mechanical properties (on a large scale) was conducted. Specifically, we inspected hydrogels of latex particles (LPs) with different chain lengths (C4, C12, C18) and studied their inner structural parameters, namely, the relationship between the density and molecular weight of crosslinking points to the mechanical strength and energy dissipation. Favorable traits of the hydrogels included compact internal structures that were basically free from defects and external structures with puncture resistance, high toughness, etc. Based on the experimental results that agreed with the theoretical results, this study provides a profound understanding of the internal structure of hydrogels, and it offers a new idea for the design of high-strength hybrid hydrogels.

## 1. Introduction

Hydrogels consisting of 3D polymer backbone networks and large amounts of water [1,2], are used in many industries for their soft and wet features [3], such as sewage treatment, contact lenses [4], sensing [5,6], separation [7,8], drug delivery [9,10], and tissue engineering [11]. Most applications require robust hydrogels that can withstand mechanical loads over time [12]. However, traditional hydrogels are exceedingly fragile because of their intrinsic inhomogeneous structure and lack of any energy dissipation mechanism [13,14]. These shortcomings severely limit the practical application of hydrogels in artificial muscles [15], cartilages [16], blood vessels [17], and tendons [18], which require toughness and load-bearing areas; therefore, it is necessary to design hydrogels with remarkable mechanical properties.

To address this need, creative strategies have been used to develop new mechanisms and unique structures to strengthen the hydrogels [19], including the pattern of double-network [20], slide-ring [21], microgel [22,23,24], hydrophobic association hydrogels [3], and nanocomposite consolidate hydrogels (NC hydrogels) [25,26]. Fu et al. successfully fabricated core-shell structured poly(stearyl methacrylate)-poly (2-acrylamido-2-methyl-1-propanesulfoni) crosslinked polyacrylamide microgels with a tensile strength of 0.3 MPa [27]. Zheng et al. demonstrated that poly (2-acrylamido-2-methylpropane sulfonate) double-network hydrogels can possess a superior mechanical characteristic of 0.35 MPa [28]. Gu et al. developed a polyacrylamide/cationic micelle double-network hydrogel. The resulting hydrogel exhibited a tensile strength of 0.36 MPa [29]. Liu et al. reported a series of hydrophobic association hydrogels containing octyl phenol polyoxyethylene (ethoxyl units were 4, 7, 10 respectively); however, the maximum tensile strengths of these gels were only 210, 203.2, and 170 KPa [3]. Later, they used palmityl alcohol poly(oxyethylene acrylate) as a hydrophobic monomer to synthesize the hydrophobic association hydrogels. The resulting gels exhibited the maximum fracture stress value of 218 KPa [3].

Generally, nanoparticle serves as an effective modifier to toughen plastics and rubber [30,31,32,33,34]. At present, plenty of researchers have fostered the hydrogels by employing nanoparticles as innovative physical cross-linkers [35,36,37,38,39,40,41]. Sun et al. [42] developed a novel nanocomposite (NC) hydrogel based on nanocrystals with a fracture stress of 229 KPa. Regretfully, these hydrogels usually exhibit a weak mechanical strength (below 0.4 MPa) after loading. Only a small amount of damage will destroy the entire network of hydrogels, they cannot immediately recover and will rapidly spread over the surrounding area through the rigid crosslinking points. The damage of NC hydrogels could hardly be repaired and recovered. There are few energy dissipation mechanisms to delay the propagation of the break. Recently, to better dissipate energy and improve the recoverability and fatigue resistance, a unique hybrid cross-linking network concept has been put forward to construct toughened NC hydrogels [43,44,45,46]. Zhao et al. fabricated NC hydrogels with excellent compression and expansion properties [47] using a hybrid crosslinking process. 

Although these prepared hydrogels exhibited improved mechanical properties, surprisingly little attention was given to the inner dissipation mechanisms [48,49]. The fundamental studies on hydrogel structures are relatively rare. To meet certain kinds of demands, a microscopic and macroscopic study of the structure and mechanical properties of hydrogels is necessary.

In this study, we developed a hydrogel with a unique pattern of hybrid latex particle structures to study the energy dissipation mechanism. In order to study the inner distribution between the polymer chains and crosslinking points of different lengths, the hydrogels were synthesized by acrylamide(AM) and hydrophobic monomers (C1, C4, C12, C18) and toughened by the different crosslinking centers which were made of SiO_2_-g-poly(butyl acrylate)-(PBA), SiO_2_-g-poly(lauryl methacrylate)-(PLA), and SiO_2_-g-poly(stearyl methacrylate)-(PSA), whose main frameworks were formed by the copolymerization of poly(acrylamide)(PAM) and hydrophobic chains. In this strategy, we primarily focused on the mechanical, rheological, and swelling properties of the hydrogels. The hybrid cross-linking structure of hydrogels could not only effectively prevent the growth of cracks but also absorb a large amount of energy. The hydrogels exhibit a high mechanical strength (0.78 MPa) and an excellent fatigue resistance. 

Most importantly, we first addressed the effect of hybrid particles of different chain lengths on the inner structures. The results show that organic/inorganic nanoparticles (NPs) as crosslinking points can effectively improve the mechanical properties of hybrid networks. In addition, we were able to gain a deeper understanding of the inner dissipation of organic-inorganic toughened hydrogels and dissipate a large number of energy under loading due to the movement and slippage of molecular chains, which can provide an insight and new inspiration for designing tough hydrogels to meet certain demands in the biomedical field. 

## 2. Materials and Methods 

### 2.1. Materials

Acrylamide (AM) and potassium persulfate (KPS) were obtained through Tianjin Fuchen Chemical Reagent Factory (Tianjin, China) and recrystallized before use. Sodium dodecyl sulfate (SDS), N,N,N’,N’-tetramethylethylenediamine (TMEDA), sodium chloride (NaCl), sodium carbonate (Na_2_CO_3_), methyl methacrylate (MMA) and butyl acrylate (BA) were supplied by Shanghai Aladdin Reagent Co. Ltd (Shanghai, China). Lauryl methacrylate (LA) and stearyl methacrylate (SA) were all purchased from Beijing Chemistry Company (Beijing, China). Colloidal silica (SiO_2_, 30 wt %) was provided by Shandong Better Company (Dongying, China). 3-Methacryloxypropyltrimethoxysilane (γ-MPS) was offered from Shanghai Meryer (Shanghai, China). Hexadecane (HD) was rendered by Acros Belgium (Shanghai, China). Acetone and ethanol were offered by Tianjin Fuchen Chemical Reagent Factory (Tianjin, China). 

### 2.2. Preparation of Core-Shell Hybrid Latex Particles

We synthesized the functionalized SiO_2_ with reference to Bourgeat-Lami [50]. Briefly, colloidal silica (75 mL), ethanol (150 mL), and SDS (150 mg) were poured into a solution, and the pH was regulated to 9.5. Following this, 9 mL γ-MPS was injected into the former system. After the edulcoration, the eventual modified SiO_2_-MPS product was obtained.

According to previous research [51], the SiO_2_-PBA was prepared as follows: the oil phase consisted of HD (1.3 g), SiO_2_-MPS (2.0 g), St (9.8 g), BA (7.2 g) and KPS (0.9 g); meanwhile, the aqueous phase was composed of SDS (0.54 g) and NaHCO_3_ (0.01 g) solutions. Then, the two phases were mixed together with electromagnetic stirring at 65 °C for 4 h. The eventual product latex with SiO_2_-g-PBA particles was obtained (Solid Content 30%). SiO_2_-g-PLA and SiO_2_-g-PSA particles were prepared using the same procedure. 

### 2.3. Synthesis of Hydrogels

We developed the hydrogels by the copolymerization of hydrophilic and hydrophobic segments, with the above-mentioned particles as a crosslinker. To be specific, one an take the P(AM/LA)-(SiO_2_-g-PBA) hydrogel as an example: the SDS (0.45 g) and NaCl (0.24 g) were blended together in deionized water (30 mL) under stirring at room temperature for 15 min to obtain a transparent solution for use. LA (0.1775 g) and SiO_2_-g-PBA nanoparticles (0.43 g) were added to the above mixture under successive stirring for 6 h. Then AM (7.5 g), 1.8 mL KPS (0.01 g∙mL^−1^) and TMEDA (10 μL) were added dropwise into the solution. Finally, the products were injected into the specified mold at 50 °C for 12 h. Two kinds of toughened hydrogels were created by the same experimental method. The first hydrogel was composed of the same hybrid particles but different hydrophobic monomers; specifically, it was defined by changing the chain lengths of the hydrophobic monomer and keeping the hybrid particles constant. For example, if (SiO_2_-g-PLA) served as the constant hybrid particles, the toughened hydrogels were defined as P(AM/MMA)-(SiO_2_-g-PLA)(C1), P(AM/BA)-(SiO_2_-g-PLA)(C4), P(AM/LA)-(SiO_2_-g-PSA)(C12), P(AM/SA)-(SiO_2_-g-PSA)(C16).

In contrast, the second hydrogel was composed of the same hydrophobic monomers but different hybrid particles; specifically, it was defined by changing the chain lengths of the hybrid particles and keeping the hydrophobic monomer constant. For instance, if P(AM/LA) served as the constant hydrophobic monomer, the toughened hydrogels were defined as P(AM/LA)-(SiO_2_-g-PBA), P(AM/LA)-(SiO_2_-g-PLA) and P(AM/LA)-(SiO_2_-g-PSA). For a comparison, the P(AM/LA) hydrogel and PAM-hybrid latex particles hydrogel were fabricated according to the same procedure mentioned above.

### 2.4. Characterization

#### 2.4.1. IR Test

The Fourier transform infrared (FTIR) spectrum was recorded in the wave-number range of 4000–500 cm^−1^ with a Nicolet 360 spectrophotometer in the spectral range of 4 cm^−1^ to 64 cm^−1^, with a resolution for each sample. Before a measurement, the latex containing LPs was freeze-dried under vacuum in a freeze (FDU-2110, EYELA, Tokyo, Japan).

#### 2.4.2. TGA Test

The thermal degradation behavior of samples was performed via a thermogravimetric analysis (TGA, Pyris-1 thermal analyzer, Perkin–Elmer, Waltham, USA) in a nitrogen environment from 50 to 600 °C (heating rate = 10 °C∙min^−1^). The specimen was purified and stored at 60 °C in a vacuum for testing. 

#### 2.4.3. DLS Measurement 

The dynamic light scattering (DLS) measurements of core-shell hybrid particles was performed on an American Brookhaven 90 Plus Particle Size Analyzer (angle setting = 90°, Brookhaven Instruments Corporation, New York, NY, USA). Each sample was tested 5 times to maintain the average particle size.

#### 2.4.4. Mechanical Measurement 

The tensile test was carried out on an equipment (SHIMADZU, model AGS-X, 100N, Kyoto, Japan) at 25 °C. All the samples were sheared into strips (length = 30 mm, width = 4 mm, thickness = 3 mm). The crosshead speed was fixed at 100 mm∙min^−1^. 

#### 2.4.5. Swelling Measurement

The hydrogel samples were conducted by being immersed into distilled water at 25 °C; the diameter and the thickness of the cylindrical as-prepared gels were 7 mm and 4 mm, and the distilled water was replaced with fresh water every day. Before the weight measurement of each swollen sample (*W_t_*), the gel samples were carefully taken out from the solution, and blotted with a filter paper to remove any free water from their surface. After the period of swelling equilibrium time, they were dried in a vacuum at 70 °C. The swelling ratio of hydrogels was calculated as follows:

(1) $$SR=\frac{\left(W_{t}-W_{dry}\right)100\%}{W_{dry}}$$

#### 2.4.6. Rheological Measurement

The rheological measurement of the hydrogel was performed by an AR 2000ex rheometer (plate parameter, *Φ* 25 mm, gap distance 2 mm, oscillatory mode, angular frequency range, 0.1-100 rad∙s^−1^, TA instruments, New Castle, DE, USA). 

## 3. Results

### 3.1. Characterization of Hybrid Latexes Particles

**Figure 1:**
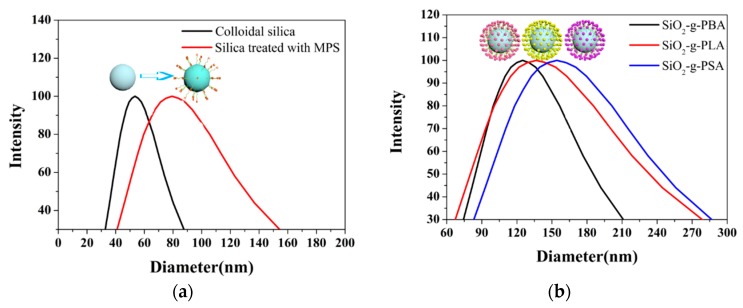
(**a**) Particle sizes and distribution of SiO_2_ and SiO_2_ treated with MPS. (**b**) Diameters of SiO_2_-g-PBA, SiO_2_-g-PLA and SiO_2_-g-PSA with different shells.

In this approach, the particle size of untreated colloidal silica and silica covered by MPS was clearly illustrated in Figure 1a; their diameters were 50 and 88 nm, respectively, meaning that the surface of SiO_2_ was successfully modified.

In order to prove that a different hydrophobic shell was grown on the surface of the SiO_2_, as can be seen in Figure 1b, the diameters of SiO_2_-g-PBA, SiO_2_-g-PLA, and SiO_2_-g-PSA were measured by DLS and found to be 125 nm, 145 nm, and 175 nm, respectively. This indicated that the hydrophobic shell was successfully grafted onto the surface of the SiO_2_ core.

### 3.2. FTIR Analysis

**Figure 2:**
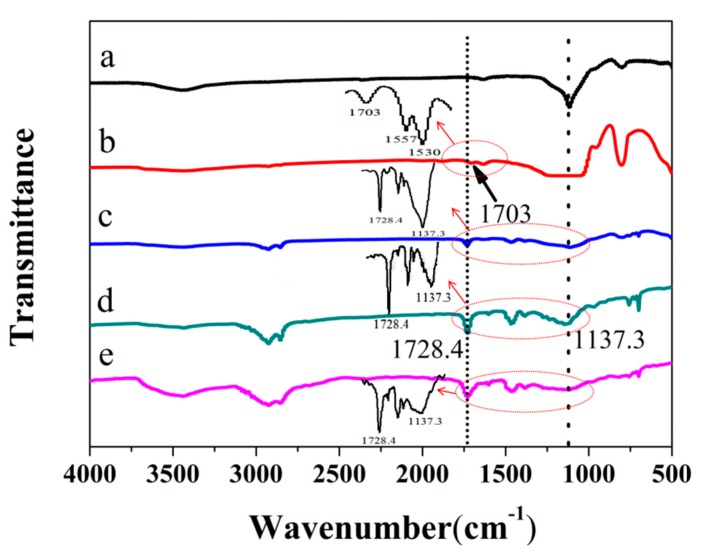
IR spectroscopy of (**a**) SiO_2_, (**b**) SiO_2_-g-MPS, (**c**) SiO_2_-g-PBA , (**d**) SiO_2_-g-PLA and (**e**) SiO_2_-g-PSA. The left inset spectra is a zoomed-in view of the corresponding area.

The structures of the samples were characterized using FTIR spectroscopy on the instrument (Nicolet, AVATAR-360, USA), and the result was shown in Figure 2: the strong band at 1000–1200 cm^−1^ was assigned to Si-O-Si bonds, and a wide peak at 3100–3700 cm^−1^ was associated with -COOH of silica. By comparison with spectrum a, the peaks emerging at 1557, 1703 and 1530 cm^−1^ were characterized by C=C, C=O and C-H stretching vibrations of γ-MPS (spectrum b), respectively. In addition, the obvious peaks at 1728.4 and 1137.3 cm^−1^ were represented as C=O groups and C-O groups of SiO_2_-g-PBA (spectrum c), SiO_2_-g-PLA (spectrum d), and SiO_2_-g-PSA (spectrum e). All of this evidence indicated that the different core-shell particles were successfully synthesized.

### 3.3. TGA Analysis

**Figure 3:**
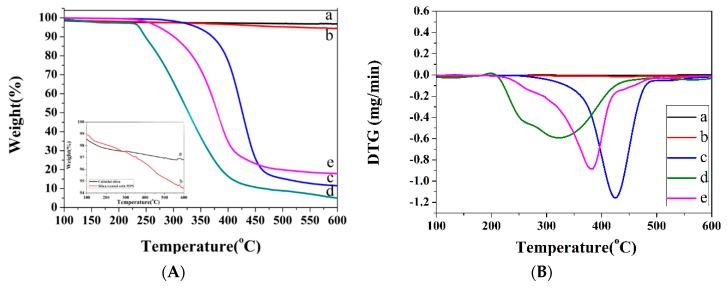
(**A**). TGA spectrum of SiO_2_ (a), SiO_2_-MPS(b), SiO_2_-g-PBA (c), SiO_2_-g-PLA (d) and SiO_2_-g-PSA particles (e); (**B**) The corresponding DTG spectrum.

Subsequently, TGA tests of pure SiO_2_ (Sample a) and SiO_2_-MPS (Sample b) were then performed, and the results are shown in Figure 3. The hydroxyl groups on the silica surface were converted to vinyl groups using γ-MPS through a condensation reaction. According to Figure 3a, the weight (wt) loss of unmodified silica and modified silica in the temperature range 100–600 °C was 3.0 wt % and 5.6 wt %, respectively. Therefore, it was apparently about 2.6 wt % of γ-MPS attached to the silica particles (Sample b). 

What is more, there was a remarkable weight loss for the SiO_2_-g-PBA (Sample c), SiO_2_-g-PLA (Sample d) and SiO_2_-g-PSA (Sample e) hybrid particles at temperatures from 200 to 430 °C, owing to the thermal decomposition of different shells on the surface of SiO_2_ (Figure 3b).

The TGA analyses of SiO_2_-g-PBA, SiO_2_-g-PLA, and SiO_2_-g-PSA were undertaken at the temperature range of 100 to 600 °C, and the results were shown in Figure 3. It can be observed that all the test samples showed similar degradation profiles. A sharp degradation between 260 and 460 °C with a 82.76 wt % loss for the PBA shell, a sharp degradation between 220 and 430 °C with a 86.6 wt % loss for the PLA shell, and a sharp degradation between 250 and 460 °C with a 76.74 wt % loss for the PSA shell was observed. Therefore, the TGA results indicated that the three different alkyl chains of PBA, PLA, and PSA were successfully grafted onto the silica particles.

### 3.4. Mechanical Properties of Hydrogels 

**Figure 4:**
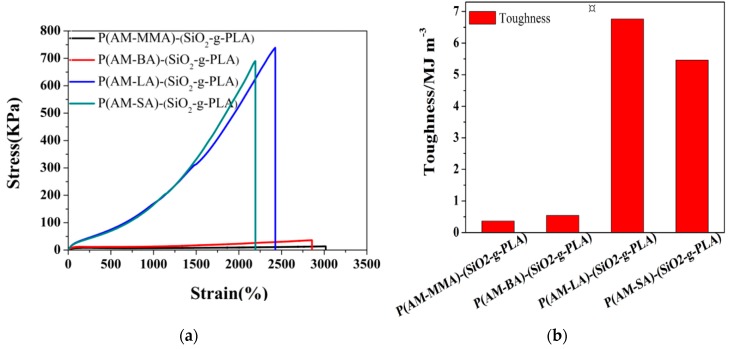
(**a**) Tensile properties of P(AM-MMA)-(SiO_2_-g-PLA), P(AM-BA)-(SiO_2_-g-PLA), P(AM-LA)-(SiO_2_-g-PLA) and P(AM-SA)-(SiO_2_-g-PLA) hydrogels. (**b**) The corresponding fracture toughness.

The mechanical properties of the hydrogels with different hydrophobic monomers (namely C1, C4, C12, C16) were conducted to quantify the specific mechanical property of the hydrogels network in Figure 4. 

In the uniaxial tensile test, the tensile stress of P(AM-LA)-(SiO_2_-g-PLA) hydrogel(0.78 MPa) was obviously higher than that of the P(AM-MMA)-(SiO_2_-g-PLA) (13.5 KPa), P(AM-BA)-(SiO_2_-g-PLA) (28.2 KPa), and P(AM-SA)-(SiO_2_-g-PLA) hydrogel (0.64 MPa) at a constant strain. The reason for the phenomenon was due to the hydrogels with different hydrophobic monomers: the prepared hydrogels exhibited an excellent mechanical strength (P(AM-LA)-(SiO_2_-g-PLA) hydrogel) based on their tight internal entanglement structure and the homogeneous distribution of crosslinking centers, which efficiently enhanced the mechanical behavior of the hydrogels. 

Moreover, the mechanical behavior of the hydrogels with MMA and BA exhibited lower stress than that of the LA-reinforced hydrogels, which indicated that the entanglement between the LPs and shorter alkyl chains was loose and unstable. This resulted in the destruction of the hydrogels. Based on the discussion above, we are convinced that it is not easy to undermine the hydrophobic groups from the steady crosslinking centers for the P(AM-LA)-(SiO_2_-g-PLA) hydrogel. 

When an external stress was applied, both of the two networks (the first network being the crosslinking of hydrophobic monomers and hybrid particles, and the second being the chemical crosslinking of AM and hydrophobic groups) used coordination interactions to redistribute the applied stress, which allowed a disentanglement from the hydrophobic segments on the HLPs, which in turn dissipated a large amount of energy. As a consequence of the coordination interactions, the hydrogels manifested an eminent tensile stress of 0.78 MPa (Figure 4a). 

Surprisingly, the mechanical strength properties of the new hydrogels decreased as the length of the hydrophobic chain increased. This meant that the hydrophobic regions with long chains possessed straight carbon chains with a larger space steric hindrance, and the long chains also tended to aggregate and blister easily. This therefore increased the number of invalid association points. All the experiments showed that the hydrogels with optimum chain lengths had much better mechanical strength properties than those without, which provided an efficient way to enhance the mechanical strength of the hydrogels.

We found that the fracture energy presented a trend of first increasing before decreasing with increasing chain lengths of the hydrophobic monomer (Figure 4b), which meant that the obtained hybrid particles could serve as energy dissipation sites (not just as simple packing sites) to regulate the dissipated energy of the inner chain by sliding synergistically with the hydrophobic monomer.

Unfortunately, comparing the stress-strain curves of the two types of hydrogels with that of a shorter chain, such as MMA and BA, showed that neither of them could act as an effective crosslinking point. Accordingly, they exhibited only 13.5 and 28.2 KPa, and were treated as a filler (Figure 4). In the uniaxial tensile test, the tensile stress of P(AM-LA)-(SiO_2_-g-PLA) was noticeably higher than that of P(AM-MMA)-(SiO_2_-g-PLA), P(AM-BA)-(SiO_2_-g-PLA), and P(AM-SA)-(SiO_2_-g-PLA), with a constant strain; this indicated that the hybrid particles could serve as energy dissipation sites (not just as simple packing sites) to regulate the dissipated energy of the inner chain by sliding synergistically with the hydrophobic monomer. All the experiments showed that the hydrogels with the optimum chain lengths have much higher mechanical properties than the others.

**Figure 5:**
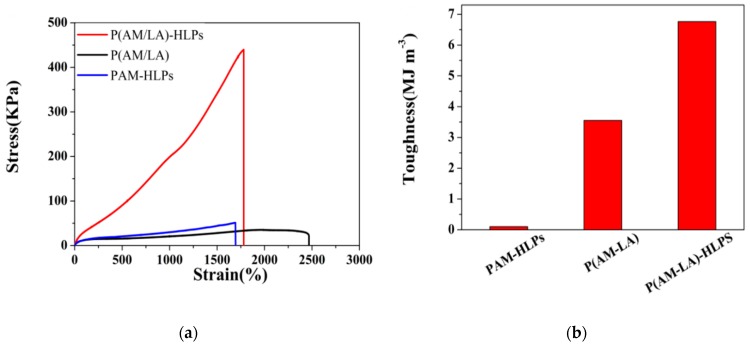
(**a**) Mechanical properties of PAM-HLPs, P(AM-LA), and P(AM-LA)-HLPs hydrogels. (**b**) The corresponding fracture toughness.

The tensile properties of three types of hydrogels were constructed in Figure 5, namely PAM-hybrid latex particles (HLPs), P(AM-LA) and P(AM-LA)-HLPs. Apparently, without hydrophobic monomers, the hybrid particles used for packing filler without the effective toughening characteristics of associated PAM hydrogels, PAM-hybrid particles, and P(AM-LA), exhibited a poor tensile stress and fracture strain, which were only 40 KPa and 1600%, and 30 KPa and 2400%, respectively. Interesting, the outstanding enhanced tensile strength (0.78 MPa) and fracture toughness (6.9 MJ∙m^−3^) of the P(AM-LA)-hybrid particle hydrogels (Figure 5b) showed, conversely, that the hydrophobic segment interacted with the hybrid particles via synergistically hydrophobic interactions.

### 3.5. Formation Mechanism of Hydrogels

**Scheme 1:**
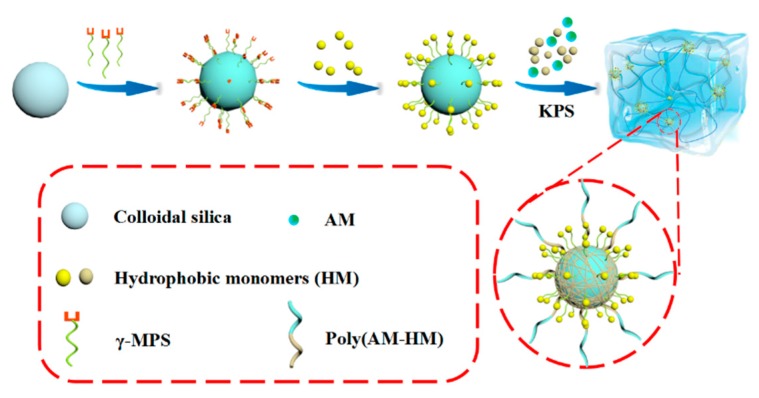
The synthesis process of hydrophobic hydrogels with inorganic-organic hybrid latex particles.

As shown in Scheme 1, the formation of hybrid hydrogels was carried out by a radical polymerization. In the reaction where hybrid NPs were dispersed uniformly in water, hydrophobic interactions caused the hydrophobic side chains to become self-assembled with hybrid particles, which formed the physical crosslinking points. From these results, the hybrid particles were shown to be vital for improving the mechanical behavior of the hydrogels, and assisted in the formation of a much more rugged framework, while also increasing the toughness and elasticity of the hydrogels.

**Figure 6:**
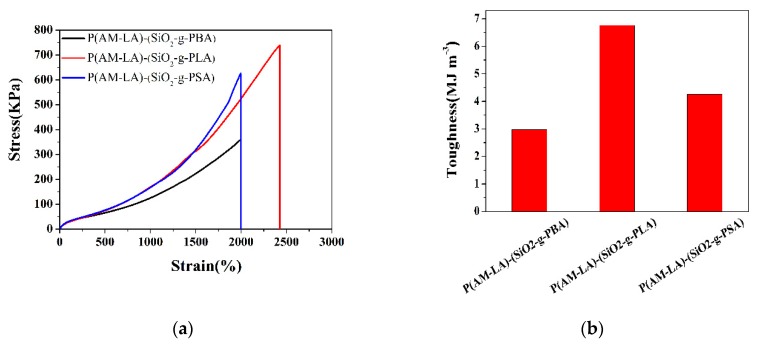
(**a**) Tensile properties of P(AM-LA)-(SiO_2_-g-PBA), P(AM-LA)-(SiO_2_-g-PLA), and P(AM-LA)-(SiO_2_-g-PSA) hydrogels. (**b**) The corresponding fracture toughness.

Therefore, we further discussed the effect of hybrid particle chain lengths on macroscopic properties, and a series of tensile tests were exhibited in Figure 6a. It could be clearly observed that the tensile stress showed a manifest trend of increasing originally, and then decreasing with the increase of the alkyl segment from the hybrid particles. It is clear that the hydrogels toughened by SiO_2_-g-PLA hybrid particles exhibited an exceeding tensile stress and fracture strain, which were superior to the others, demonstrating that the hybrid particles served as an effective physical crosslinker with an optimum chain length of C12 in the hydrogels preparation. At the moment, they were assisted in the formation of a much more vigorous framework and generated the increased toughness and stretchability of the hydrogels.

In comparison with the theories raised by Liu [3], the converse phenomenon was displayed in Figure 6, due to a strong association effect between the hydrophobic monomer and hybrid particles: the long chain cannot weaken the formed association but rather leads to a strengthening of the mechanical properties. They can interact with each other in a specific way. Under the external force, the hybrid particles could show an enormous elastic deformation like a rubber particle, play the roles of both physical hydrophobic entanglement zones and rubber toughening zones, and change the dissipated energy of their internal structure with the elastic deformation.

Unfortunately, the toughness conversely decreased gradually when we further increased the chain lengths of the hybrid particles, which meant that the hybrid particles with excessively long chains were difficult to disperse homogeneously in the polymer structural matrix. The inhomogeneous entanglement of the long chains around the surface of the hybrid particles hindered the effective association between the hydrophobic monomer and hybrid particles, which resulted in an uneven distribution of stress and a decrease in toughness.

As observed, it is important that the hydrophobic segments and hybrid particles with proper lengths could form the effective hydrophobic association centers which dissipate a large amount of energy. Thus, the P(AM-LA)-(SiO_2_-g-PLA) hydrogels with a moderate chain-length exhibited a significant improvement in mechanical strength (Figure 6b).

### 3.6. Rheology Properties of Hydrogels

**Figure 7:**
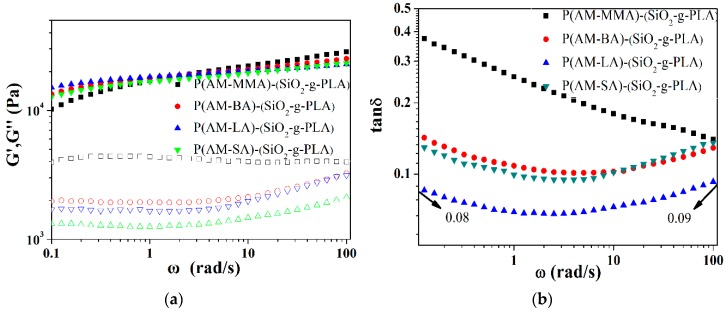
(**a**) Storage modulus (*G’*) and loss modulus (*G’’*) of P(AM-MMA)-(SiO_2_-g-PLA), P(AM-BA)-(SiO_2_-g-PLA), P(AM-LA)-(SiO_2_-g-PLA) and P(AM-SA)-(SiO_2_-g-PLA) hydrogels. (**b**) The relationship between the loss factor (*tanδ*) and frequency of P(AM-MMA)-(SiO_2_-g-PLA), P(AM-BA)-(SiO_2_-g-PLA), P(AM-LA)-(SiO_2_-g-PLA) and P(AM-SA)-(SiO_2_-g-PLA) hydrogels.

In order to reveal the connection between microstructure and macroscopic mechanical properties, a rheological test was carried out. The storage modulus (*G’*) and loss modulus (*G’’*) of four hydrogels with different hydrophobic groups were presented at different frequencies, to describe the inner crosslinking density and structure of the hydrogels (Figure 7a). With regard to the hydrogels with different hydrophobic chains, the *G’* value was almost the same, while the *G’’* value varied with an increase of frequency, which indicated that all the gels were formed by physical crosslinking microregions.

What is more, one interesting phenomenon was that the rheological measurements showed that the P(AM-LA)-hybrid particles hydrogels were independent of the *ω*. It was found that the network of the experimental hydrogels was stable, possessed a more effective crosslinking density, and had a more optimal structure. In the ideal case, the crosslinking point composed of hydrophobic monomers and latex spheres was a tight and dense association of microdomains. Conversely, for the P(AM-MMA)-hybrid particle, P(AM-BA)-hybrid particles, and P(AM-SA)-hybrid particles hydrogels, the lengths of the hydrophobic molecular chains were different, and the *G’* increased with a high *ω*, indicating that it probably had many weakly associated microdomains; thus, the corresponding internal network structure was also loose and inhomogeneous, which caused the network to store less energy.

As discussed above, only a moderate structure of hydrogels was found to be endowed with a lower *G’* and *G’’*, and with an appropriate crosslinking density for the polymer network. In brief, the tight compact entanglement structure between the resulting polymer molecular chains and the homogeneous distribution of crosslinking centers made the network store more energy and possessed the best mechanical properties.

Nevertheless, we further performed research to explore the relationship between the loss factor (*tanδ*) and the frequency, as shown in Figure 7b. Somewhat surprisingly, as the hydrophobic alkyl side chain of hydrogel increased, the *tanδ* of four hydrogels exhibited a down-up tendency. In contrast, we discovered that the *tanδ* of the P(AM-LA)-(SiO_2_-g-PLA) hybrid hydrogels were found to be at the same level. Taken together, this showed that the hydrogels contained very few defects, resulting in an excellent elasticity.

### 3.7. Swelling Properties of Hydrogels

**Figure 8:**
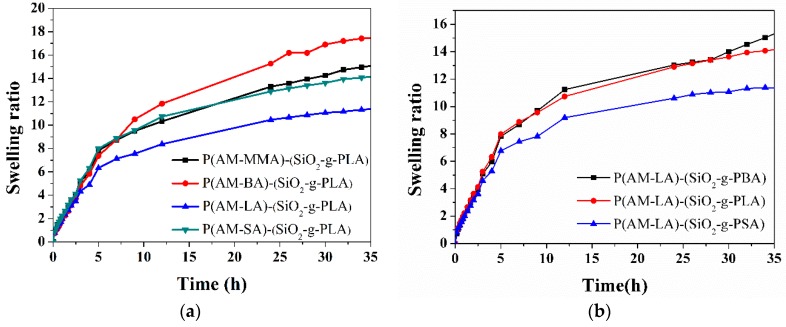
(**a**) The relationship between the swelling ratio and swelling time of the P(AM-MMA)-(SiO_2_-g-PLA), P(AM-BA)-(SiO_2_-g-PLA), P(AM-LA)-(SiO_2_-g-PLA) and P(AM-SA)-(SiO_2_-g-PLA) hydrogels. (**b**) The relationship between the swelling ratio and swelling time of the P(AM-LA)-(SiO_2_-g-PBA), P(AM-LA)-(SiO_2_-g-PLA) and P(AM-LA)-(SiO_2_-g-PSA) hydrogels.

To examine whether the incorporation of hydrophobic segments and hybrid particles would affect the swelling ratio, a few tests were carried out in Figure 8a. Clearly, it was found that hydrogels with different hydrophobic chains had significantly different swelling ratios. As described above, the hydrogels with short hydrophobic chains, like MMA and BA, were featured with a high swelling ratio and an uncompact polymeric network structure. As a result, the shorter the length of the molecular chains, the looser the structure and lower the cross-linking density. However, it is noteworthy that the increase of the chain lengths of the hydrophobic monomer decreased the swelling ratio, which caused the network structure to become even more dense and compact, which in turn considerably inhibited the gel from swelling; as a result of this, the lifetime of hydrogels in the aqueous solution decreased slowly. It is evident that the hydrophobic association effect existed indeed. 

The swelling ratio of the P(AM-LA)-(SiO_2_-g-PBA), P(AM-LA)-(SiO_2_-g-PLA) and P(AM-LA)-(SiO_2_-g-PSA) hydrogels were tested in Figure 8b. Obviously, all the curves rose initially but finally became stable. One phenomenon was that, in general, the P(AM-LA)-(SiO_2_-g-PLA) hydrogels exhibited a lower SR than the others. Based on the above analysis, a possible reason was that the P(AM-LA)-(SiO_2_-g-PLA) hydrogel had a higher compactness of network, which was assisted in the previous tensile performance results.

### 3.8. Parameters of the Network Structure of the Hydrogels 

In this work, the effective crosslinking density(*ν_e_*) of the hybrid hydrogels is acquired by Equation (2) [52,53,54].

(2) $$G'\approx G=\left(1-\frac{2}{\Phi }\right)RTv_{e}v_{2}{}^{\frac{2}{3}}$$

where *ν*_2_ is the volume fraction of the polymer of swollen hydrogels [53,54], *R* denotes a gas constant, and *Φ* is 4. *ν*_2_ is obtained from Equation (3) [53,54,55]:

(3) $$v_{2}={\left[1+\frac{\left(q_{F}-1\right)\rho }{d}\right]}^{-1}$$

where *q_F_* presents *W_t_/W_dry_*, *ρ* denotes the density of the polymer, and *d* is 1.00 g∙mL^−1^. After merging Equations (2) and (3), the reckoned *ν_e_* are displayed in Table 1.

**Figure 9:**
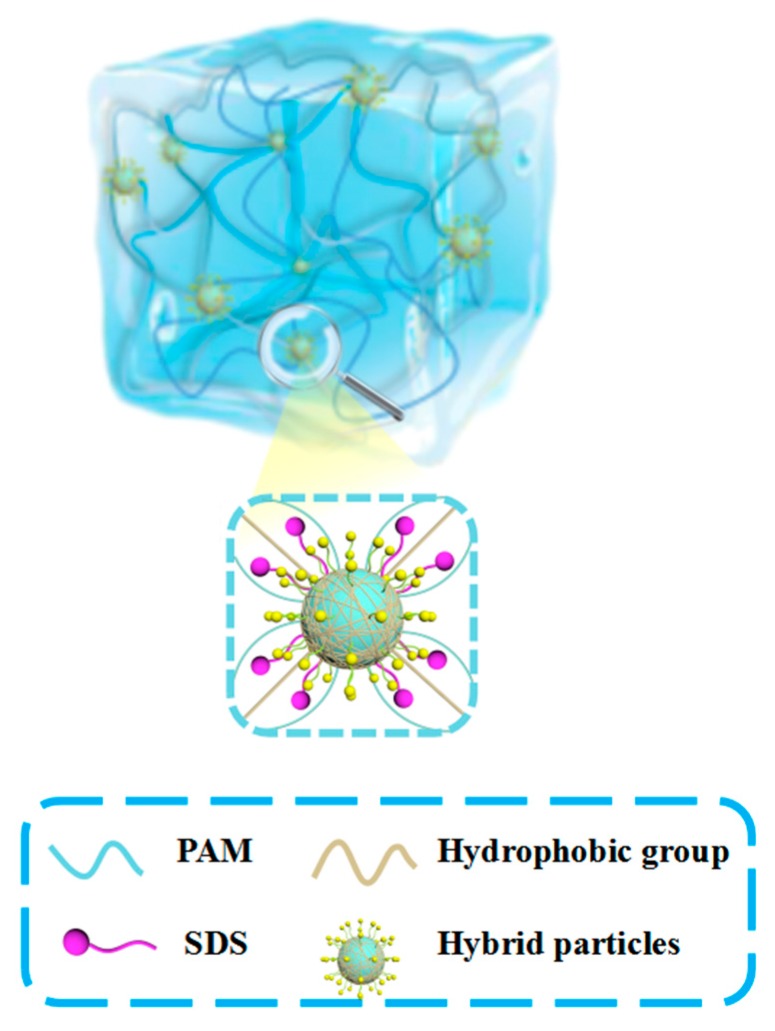
Diagram of the inner micellar structure of hybrid hydrogels.

Adhering to rigorous principles, we must take the internal structure in different periods into consideration; we therefore verify *ν*_0_ (effective crosslinking density), and *M_c_* (the average molecular weight between the cross-linkage center) in another way according to the rubber elastic theory. Herein, *ν*_0_ is obtained as follows [3]:

(4) $$\sigma =v_{0}kT(\lambda -\lambda^{-2})$$

where *σ* denotes the force per unit original cross-sectional area of the gel network, *λ* represents the relative extension, and *k* is Boltzmann’s constant [3]. *M_c_* can calculated from the Equation (5) [3]:

(5) $$v_{0}=\frac{\rho N_{A}}{M_{c}}(1-\frac{2M_{c}}{M_{n}})$$

where *ρ* is the density of the polymer, *N_A_* is 6.02 × 10^23^, and *M_n_* is the molecular weight of the primary molecular chains [3]. Clearly, an increase in *ν*_0_ is always accompanied by a decrease in *M_c_* in Table 1, and we speculate the best micellar structure via the Equation (6) [3]:

(6) $$N_{H}=\frac{\left[M_{H}\right]N_{agg}}{\left[S\right]-cmc}$$

where *N_H_* is the number of hydrophobic groups in the hydrophobic micelles, and where [*S*] and [*M_H_*] represent the SDS and the initial molar concentration, respectively. The *N_H_* value is about 29. Consequently, we predict the distribution pattern in a micelle with a ratio of 1:2 of hydrophobic units and surfactant in Figure 9 (*N_agg_* = 60). 

We interpret the relationships of latex particles and the hydrophobic micelles, and calculate the number of latex particles per unit volume based on the equation [19]:

(7) $$\nu_{p}=\frac{6M_{n}\chi }{\pi \varphi_{p}D_{v}^{3}}$$

where *M_n_* represents the latex particles concentration, and *χ* is the conversion rate. *Φ_p_* and *D_v_* denote the system density and average size of latex particles [19]. By calculation, the data are showed in Table 2.

Herein, we can also achieve *χ_c_* (the number of micelles per unit volume) by the following equation [26]:

(8) $$\chi_{c}=\frac{\left(\frac{\left[SDS\right]}{N_{agg}}-CMC\right)*n}{V}$$

Based on the above results, the *χ_c_* was certain (1.632 × 10^17^) when the hybrid particles were changed. However, the number of latex particles decreased with the increase of the chain lengths of hybrid particles (Figure S1), implying that the effective association was more important than the number of latex particle as well as its chain length and size.

Combined with the previous analysis, the P(AM-LA)-hybrid particles hydrogel had the maximum tensile stress and the most compact entanglement. The homogeneous distribution of polymer molecular chains and crosslinking centers allowed the network to store more energy and obtain the best association. As a result, the theoretical calculation data was consistent with that of an actual experiment (Supporting information).

### 3.9. Self-Recovery of HA-Gels 

**Figure 10:**
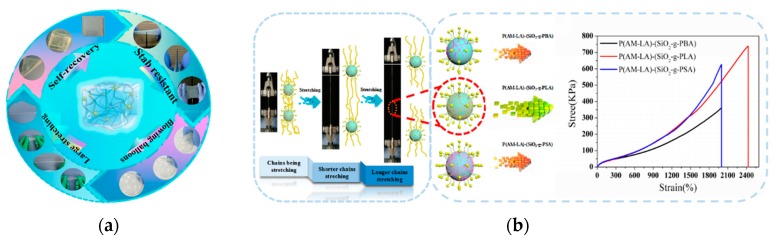
(**a**) A display of the mechanical properties of hybrid hydrogels: shape memory, knotting (Videos S1 and S2, the working length of the screwdrivers was 100 mm, the working size of the screwdrivers: 15 mm in the blade width and 5 mm in thickness), stretching, and blowing balloons (Video S3, the maximum diameter size of the fully dilated balloon was 20 cm); the water content of all the hydrogels mentioned above (shape memory, knotting, stretching, and blowing balloons) was 76.5%; (**b**) The energy dissipated mechanism of hybrid hydrogels.

As shown, it was notable that, upon being damaged, the hydrogels could heal and recover well; their molecular chains kept slipping and curling and drumming into balloon (Figure 10a, Video S1–S3). Additionally, the samples soon exhibited self-recovery, like rubber [27,56,57], when the hydrogel was folded. Obviously, when an external tension force was loaded on the hydrogel, they dissipated a multitude of energy via the deformation between the hydrophobic segments and hybrid particles along the external force direction (Figure 10b). When the hydrogel was in a large deformation, the physical cross-linking structures were destroyed and spontaneously recombined. Thereby, the superior mechanical properties of hydrogels could be ascribed to a reinforcing mechanism, insofar as inorganic-organic hybrid particles were employed as effective rubber toughening agents.

## 4. Conclusions

In this investigation, we proposed a straightforward method to effectively create toughened and ultra-elastic hydrogels with inorganic-organic hybrid particles. Due to the unique reversible network structures, the physically dynamic crosslinking between the hybrid particles and hydrophobic segments under an external force could undergo a reversible disentanglement to alleviate a large amount of energy. Therefore, these obtained hydrogels had quick self-recovery and puncture-resistance properties, and their mechanical properties could also be tuned by the inner structures. This study primarily addressed the connection between the inner microstructure network and the mechanical properties of the hybrid hydrogels and inner dissipation mechanism. We believe that by exploring the exact energy dissipation mechanism of enhanced rubber theory, this study broadens opportunities for developing soft and tough materials. In addition, the proposed synthesis method could be a promising candidate for the construction of new-generation toughened hydrogels.

## Figures and Tables

**Table 1:** Network structure parameters of hybrid hydrogels.

Sample	*ρ* (g/mL)	*q_F_*	*υ*_2_	*G’* (*ω* = 1 rad/s)	*υ_e_* (mol/m^3^)	*υ*_0_	*M_C_*
P(AM-MMA)-(SiO_2_-g-PLA)	1.2923	4.066	0.2015	10180	22.06	8.38	11.08
P(AM-BA)-(SiO_2_-g-PLA)	1.2933	3.700	0.2226	13380	27.13	13.69	10.66
P(AM-LA)-(SiO_2_-g-PLA)	1.2959	3.466	0.2383	15070	29.19	32.95	8.88
P(AM-SA)-(SiO_2_-g-PLA)	1.2979	4.130	0.1975	12720	27.94	31.12	11.75
Pure PAM	1.1890	3.758	0.2337	6400	12.81 ^a^	-	-

^a^ It is obtained in paper [56].

**Table 2:** Latex particles and micelles of the hydrogels.

Sample	*M_n_*
P(AM-LA)-(SiO_2_-g-PBA)	1.15 × 10^17^
P(AM-LA)-(SiO_2_-g-PLA)	8.2 × 10^16^
P(AM-LA)-(SiO_2_-g-PSA)	4.9 × 10^16^

## Supplementary Materials

The following are available online at http://www.mdpi.com/2073-4360/11/2/352/s1. Figure S1: Diagram of inner micellar structure of hybrid hydrogels with different LPs, Video S1: Display of stabbing of hybrid hydrogels (on the front view), Video S2: Display of stabbing of hybrid hydrogels (on the broadside view), Video S3: Display of blowing balloons of hybrid hydrogels.
