# Supplementary material for: Tuning Physical Crosslinks in Hybrid Hydrogels for Network Structure Analysis and Mechanical Reinforcement
Source: Polymers (Basel). 2019 Feb 18;11(2):352. doi: 10.3390/polym11020352 (PMC6419201; doi:10.3390/polym11020352)
Supplement: Supplementary file 1 [file polymers-11-00352-s001.zip › polymers-433604-SI/Supporting Information.pdf]

polymers

Supporting Information

Tuning Physical Crosslinks in Hybrid Hydrogels for Network Structure Analysis and Mechanical Reinforcement

Xue Lv, Chuang Liu, Zhubao Shao*and Shulin Sun*

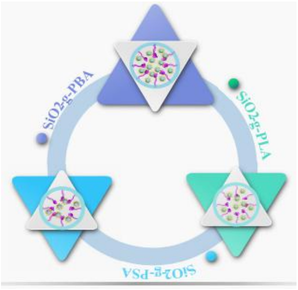

Figure S1 Diagram of inner micellar structure of hybrid hydrogels with different LPs.

As illustrated in Table 2 in the main text, in the system, the number of micelles per unit volume (χ_c) is 1.632×10^{17}. The number of micelles in the hydrogel was certain while the numbers of hybrid particles was changed. However, the number of latex particles decreased with the increase of the chain length of hybrid particles(Figure S1), implying that the effective association was more important than the number of latex particle as well as its chain length and size.

It is noting that the puncture resistant properties of hybrid hydrogels and no damage after stabbing the samples in video S1-S2, and interesting that the hydrogels membrane like rubber, it can drum into a big balloon and then frustrated to its initial shape soon in video 3. The above exhibitions illustrates the excellent self-healed and self-recovery of hybrid hydrogels.

Video S1, S2, S3.
