# Supplementary figures and images for: Tuning Physical Crosslinks in Hybrid Hydrogels for Network Structure Analysis and Mechanical Reinforcement
Source: Polymers (Basel). 2019 Feb 18;11(2):352. doi: 10.3390/polym11020352 (PMC6419201; doi:10.3390/polym11020352)

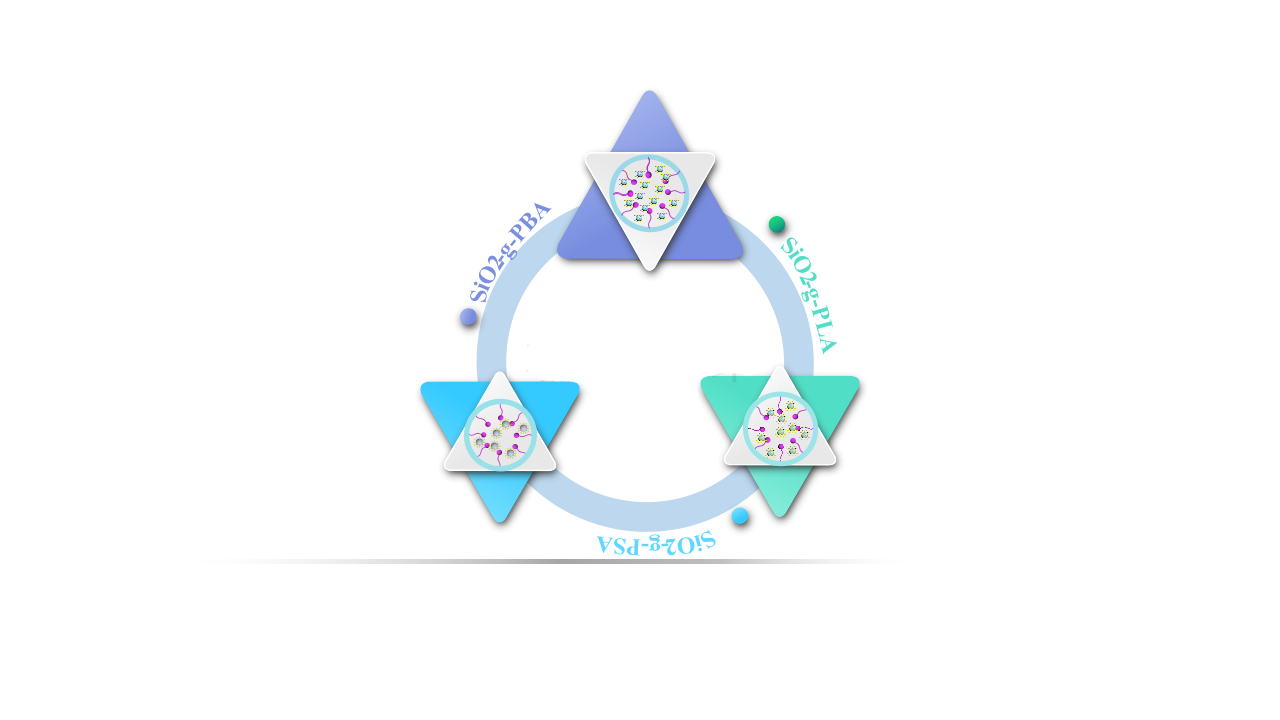

Supplement: Supplementary file 1 [file polymers-11-00352-s001.zip › polymers-433604-SI/FIGURE s1.tif]
